# Curcumin nanoparticles: physico-chemical fabrication and its in vitro efficacy against human pathogens

**DOI:** 10.1007/s13205-015-0302-9

**Published:** 2015-05-07

**Authors:** Raksha S. Pandit, Swapnil C. Gaikwad, Gauravi A. Agarkar, Aniket K. Gade, Mahendra Rai

**Affiliations:** Department of Biotechnology, S.G.B. Amravati University, Amravati, Maharashtra 444 602 India

**Keywords:** Antibacterial, Curcumin nanoparticles, Curcumin nanoparticles cream, Bacterial infections, Human pathogens

## Abstract

Curcumin is one of the polyphenols, which has been known for its medicinal use since long time. Curcumin shows poor solubility and low absorption, and therefore, its use as nanoparticles is beneficial due to their greater solubility and absorption. The main aim of the present study was the formation of curcumin nanoparticles (Nano curcu), evaluation of their antibacterial activity against human pathogenic bacteria and formulation of Nano curcu-based cream. We synthesized Nano curcu by sonication method. The synthesis of Nano curcu was assessed for their solubility in water and by UV–visible spectrophotometry. Further, the nanoparticles were characterized by Fourier transform infrared spectroscopy, transmission electron microscopy, nanoparticle tracking and analysis, and zeta potential analysis. In vitro antibacterial activity of Nano curcu was evaluated against *Escherichia coli*, *Staphylococcus aureus*, and *Pseudomonas aeruginosa*. The cream containing Nano curcu was found to be effective against human bacterial pathogens and hence can be used for treatment of bacterial diseases.

## Introduction

*Curcuma longa* L. is a perennial herb, which belongs to family Zingiberaceae, and commonly known as turmeric. It occurs in tropical and sub-tropical regions throughout the world. It is commonly cultivated in Asian countries, mostly in India and China and is extensively used in ayurveda, unani, and siddha systems of medicine as one of the household therapies to alleviate different diseases (Araujo and Leon 2000; Chattopadhyay et al. 2004; Nawaz et al. 2011). Curcumin suppresses the activity of many bacteria such as *Staphylococcus aureus*, *Salmonella paratyphi* (Chaudhary and Sekhhon 2012) and *Bacillus subtilis*, *B. macerans, B. licheniformis*, and *Azotobacter* (Naz et al. 2010). Curcumin is also found to be effective against 20 types of *Candida* species (Martins et al. 2009). It has been observed by trials on human and mouse that oral consumption of curcumin shows less bioavailability and it undergoes intestinal metabolism (Sharma et al. 2005; Anand et al. 2007). These obstacles of curcumin can be eliminated by synthesis of curcumin nanoparticles (Nano curcu), liposomes, micelles, and phospholipid complexes which can be used for the purpose of longer circulation, permeability and increased resistance to metabolic processes (Aggarwal et al. 2006; Nawaz et al. 2011; Ravichandran 2013). Curcumin loaded in poly (lactic-coglycolic acid) (PLGA) nanospheres was synthesized by using solid/oil/water emulsion solvent evaporation technique (Mukerjee and Vishwantha 2009). PLGA-loaded Nano curcu are one of the efficient tools which can be used in the cancer therapy (Mukerjee and Vishwantha 2009). Curcumin-loaded hydrogel nanoparticles can act as an adjuvant in malarial treatment which reduces the use of antimalarial drugs (Dandekar et al. 2010).

Polymeric synthesis of curcumin-loaded nanoparticles was found effective against malignant brain tumors by inhibiting the growth of brain tumor cells (Lim et al. 2011). In vitro study of synthesized nanoparticles by fatty acid coacervation technique revealed that they are effective in the treatment of cancer (Chirio et al. 2011). Also, curcumin-loaded PLGA nanoparticles were found to be novel candidates in the treatment of ovarian cancer (Yallapu et al. 2010). It was demonstrated from the study tha Nano curcu can stop the primary stage of metastasis, and therefore, it can be used as one of the novel treatments in cancer (Bisht et al. 2007). It was reported that the synthesis of solid lipid Nano curcu was useful to enhance the oral bioavailability of curcumin (Bansal and Munjal 2011). Antibacterial activity of Nano curcu was tested against different types of Gram-positive and Gram-negative bacteria (Bhavana et al. 2011). Silver nanocomposite film of curcumin was very effective material for antibacterial application (Varaprasad et al. 2011). The researchers have also prepared sodium carboxylmethyl cellulose (SCMC) silver nanocomposite films (Varaprasad et al. 2011). Curcumin chitosan-poly (vinyl alcohol)-silver nanocomposite film was prepared by Vimla et al. (2011) in order to increase applications as antibacterial packaging, wound dressing, and antibacterial materials. Different nanoparticles like curcumin, silver, and chitosan nanoparticles were synthesized and it was found that Nano curcu along with silver and chitosan nanoparticles showed anti-parasitic activity against *Giardia lamblia* (Said et al. 2012). Curcumin albumin nanoparticles were formulated by using desolvation method which is one of the novel methods of nanoparticles synthesis and albumin nanoparticles are one of the promising tools for increasing the action of curcumin as a drug (Jithan et al. 2011).

In the present study, we report physico-chemical synthesis of Nano curcu, their formulation and activity against *Escherichia coli*, *Staphylococcus aureus*, and *Pseudomonas aeruginosa*. The formulated cream is a new generation of antiseptic cream which could be used against dermatological infections.

## Materials and methods

Curcumin [1,7-bis (4-hydroxy-3-methoxyphenyl)-1,6-heptadiene-3,5-dione] powder was procured from Hi-Media Laboratories Ltd., Mumbai.

### Synthesis of Nano curcu

Stock of curcumin solution (5 mg/ml) was prepared by dissolving curcumin powder in Dichloromethane (20 ml). One ml of stock solution was added to boiling water (50 ml) in drop-wise manner under ultrasonication condition with an ultrasonic power and frequency of 50 kHz. The solution was sonicated for about 30 min. After sonication, the mixture was stirred at 800 rpm for about 20 min till the orange colored precipitate was obtained. Thereafter, supernatant was discarded and the pellet obtained was used for further study.

### Preliminary detection of Nano curcu by UV–visible spectroscopy

The preliminary detection of synthesized Nano curcu was carried out by UV–visible spectrophotometer (Shimadzu UV-1700, Japan), scanning the absorbance spectra in the range of 200–800 nm wavelength.

### Characterization of Nano curcu

#### Nanoparticle tracking analysis system (NTA)

To determine average size and size distribution of Nano curcu, NTA analysis was performed by LM 20 (Nanosight Pvt. Ltd., UK). Liquid sample of Nano curcu was introduced into a scattering cell through which a laser beam (approx. 40 mW at *k* = 635 nm) was passed. Particles present within the path of the laser beam were observed via a dedicated non-microscope optical instrument LM 20 having CCD camera. The motion of the particles in the field of view (approx. 100 × 100 μm) was recorded (at 30 fps), and from the subsequent video and images, size of particles was calculated.

#### Fourier transform infrared spectroscopy (FTIR)

Nano curcu were characterized by FTIR (Perkin-Elmer FTIR-1600, USA) mixing dried powder of nanoparticles with KBr. Spectra were taken in the range of 500–2000 cm^−1^ at a resolution of 4 cm^−1^. The data of FTIR reveal information about functional groups which are present in the Nano curcu.

#### Zeta potential measurement

Zeta potential measurements of synthesized nanoparticles were performed with Zetasizer Nano ZS 90 (Malvern Instrument ltd, UK) by using zeta dip cells. Zeta potential of synthesized nanoparticles was analyzed to determine the charges present on the surface of nanoparticles and its stability at pH 7. The samples for analysis were prepared by mixing of Nano curcu colloid in dichloromethane in 1:10 proportion. For measuring zeta potential, 1000 µl of the sample was taken in clear disposable zeta cells.

#### Transmission electron microscopy

Transmission electron microscopic analysis (TEM) was useful to determine the size and topology of synthesized Nano curcu. A drop of solution containing Nano curcu was placed on the carbon-coated copper grids and kept in infrared light until sample gets dried. After drying, powder of nanoparticles was loaded on specimen holder. TEM micrographs were taken by analyzing the prepared grids on Philips CM 200 super twin’s TEM operating at 200 kV (0.23 nm resolution) instrument.

#### X-ray diffraction method

X-ray diffraction is the method to determine crystalline structure or phase of crystal. Dried Nano curcu powder was used for analysis purpose. The diffraction patterns were recorded by PAN analytical X PRT PRO, D-8, Advanced Brucker instrument (The Netherlands).

### In vitro evaluation of antibacterial activity of Nano curcu

#### Test bacteria

*Escherichia coli* (ATCC 14948) and *S. aureus* (ATCC 333591) were procured from American Type Culture Collection Center, USA, and *P. aeruginosa* (MTCC 4676) from Microbial Type Culture Collection, Chandigarh. The antibacterial activity of the Nano curcu and silver nanoparticles was assessed against test bacteria by using Kierby–Bauer disc diffusion method (Bauer et al. 1960). Antibiotics chloramphenicol and gentamycin were used as standard, while performing antibacterial activity of nanoparticles. These plates were then incubated at 37 °C for 24 h. Zones of inhibition were measured.

### Formulation of antibacterial cream

It was performed in two phases, that is, in water and oil phase. In water phase, 40 ml of distilled water was taken in beaker. Then, cetostearyl alcohol (8 g) was added and it was heated slightly to dissolve completely. It was followed by addition of methyl paraben (0.3 g) and dissolved by heating. Then, 30 g of glycerol was added followed by tween 80 (3.6 g). All the above components were mixed together (except drug) which results in the formation of water phase.

For oil phase, liquid paraffin (5 g) was taken in a separate beaker and it was heated. Then, white paraffin (10 g) was taken in another beaker and heated later, and liquid paraffin was mixed with it which results in the formation of oil phase. After the formation of both the phases, they were mixed together drop by drop with continuous stirring. After mixing of both the phases, Nano curcu (5 mg ml^−1^) were added to the mixture by continuous stirring on magnetic stirrer to dissolve the drug properly.

## Results

**Fig. 1:**
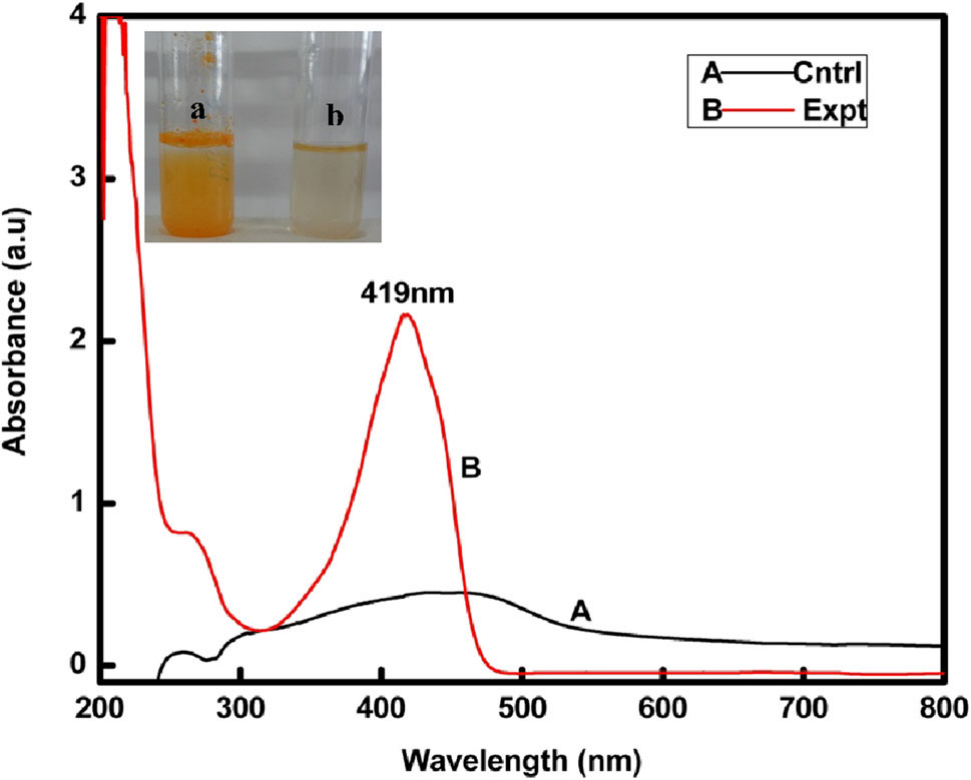
UV–vis spectra of synthesized Nano curcu. (Spectra A-curcumin powder in dichloromethane, Spectra B-Nano curcu). Inset fig comparative solubility of curcumin powder and Nano curcu. *a* Curcumin in water, *b* Nano curcu in water

**Fig. 2:**
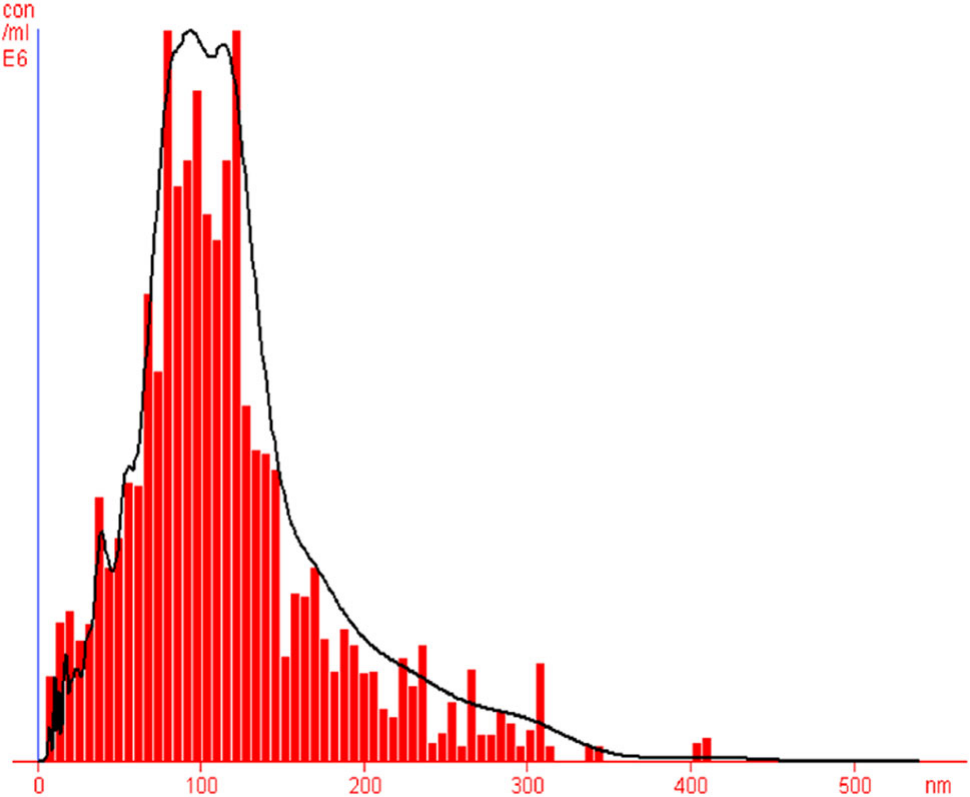
Nanoparticles tracking and analysis system (NTA) shows the size of Nano curcu

**Fig. 3:**
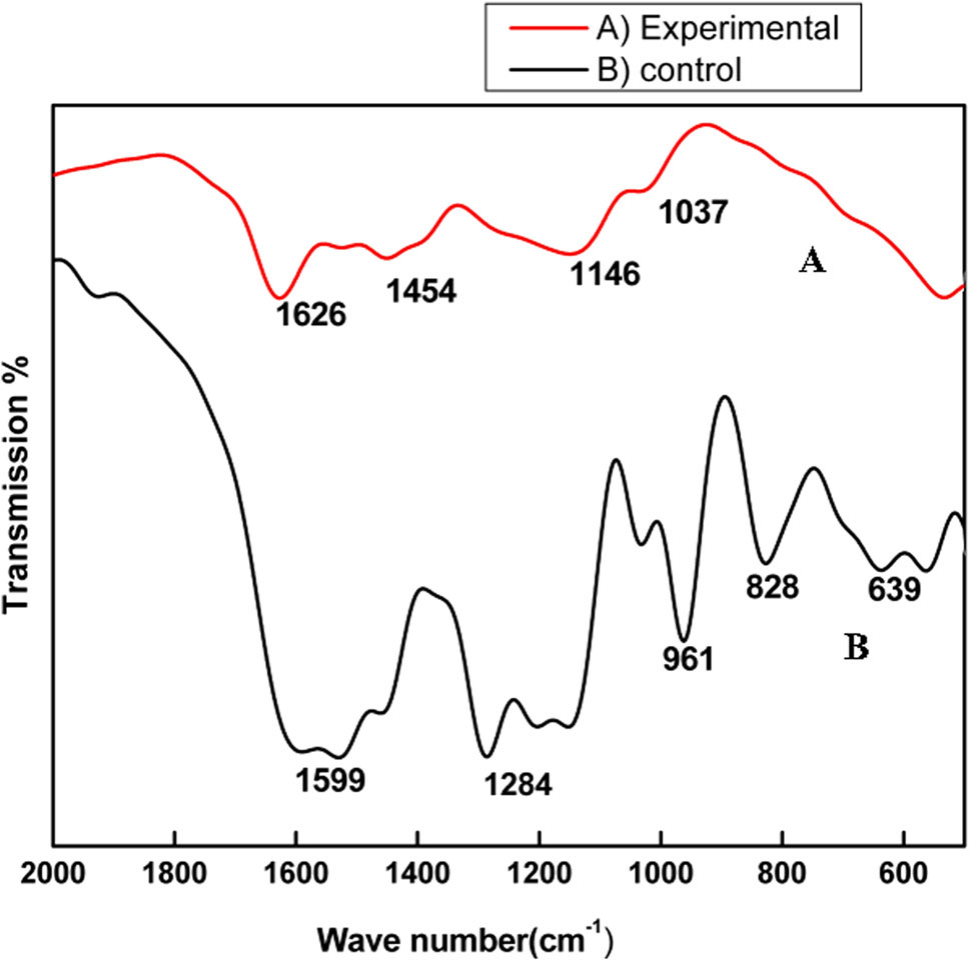
FTIR spectra of Nano curcu. *a* Experimental-Nano curcu, *b* control-curcumin in dichloromethane

**Fig. 4:**
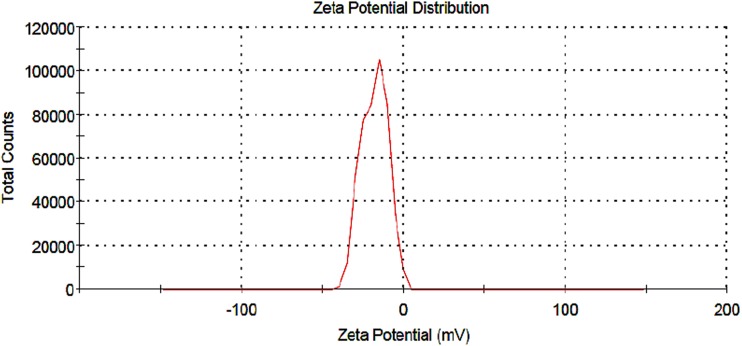
Zeta potential measurement of Nano curcu (−18 mV)

**Fig. 5:**
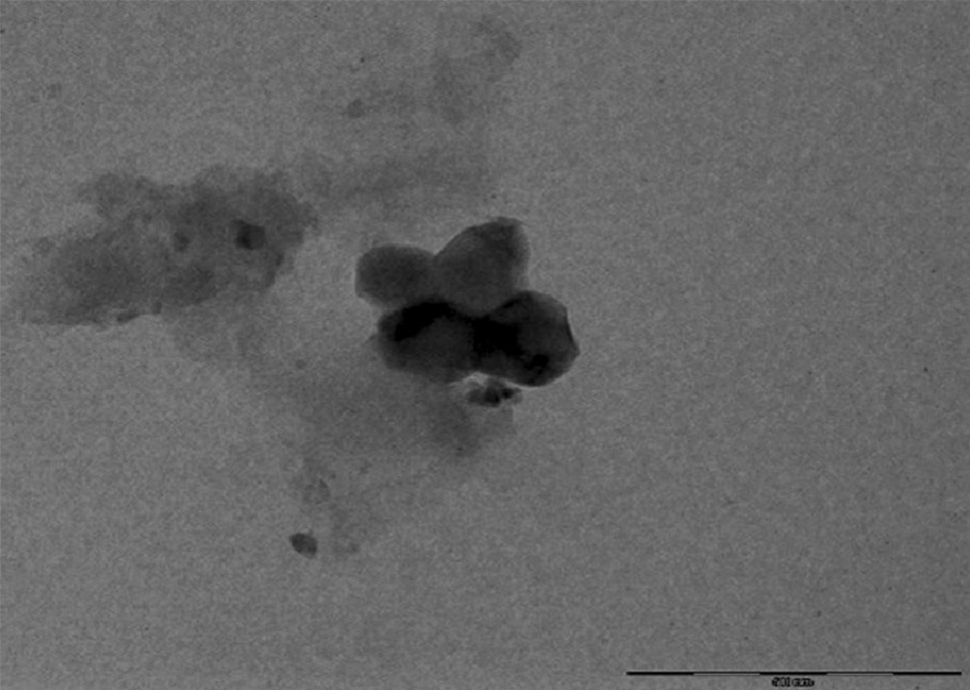
TEM image showing size and shape of Nano curcu

**Fig. 6:**
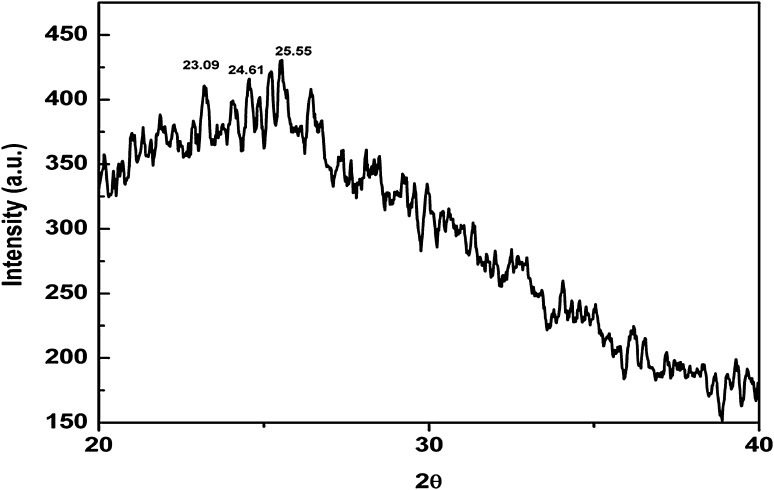
X-ray diffraction pattern of synthesized Nano curcu

Nano curcu detection was done by UV–visible spectroscopy and it was scanned in the range of 200–800 nm. The absorption spectra of curcumin in dichloromethane (control) and synthesized Nano curcu showed absorbance peak at 419 nm which is the characteristic feature of Nano curcu (Fig. 1). Further characterization of Nano curcu was performed by nanoparticles tracking and analysis system (NTA) to determine the average size and particle size distribution. From the NTA analysis, the mode value was found to be 92 nm, with the average size of 110 nm (Fig. 2). FTIR spectrum of curcumin in dichloromethane (control) and Nano curcu (experimental) was recorded. In FTIR spectrum of Nano curcu, peaks were observed at 1626, 1454, 1146 and 1037 cm^−1^ (Fig. 3). The zeta potential of Nano curcu was found to be −18 mV which showed moderate stability of Nano curcu (Fig. 4). The magnitude of the zeta potential gives an indication of the potential stability of the colloidal system. It is the potential which is measured, when one measures the velocity of the particles in a D.C. electric field. TEM analysis was performed to determine the size and shape of nanoparticles. It was found that Nano curcu showed spherical shape and polydisperse particles having the size range of 60–80 nm (Fig. 5). X-ray diffraction analyses are applied to determine the crystalline nature of Nano curcu. X-ray diffractograms of synthesized Nano curcu showed characteristic peaks at diffraction angle of 2*θ* at 23.03, 24.60, and 25.55 (Fig. 6).

**Fig. 7:**
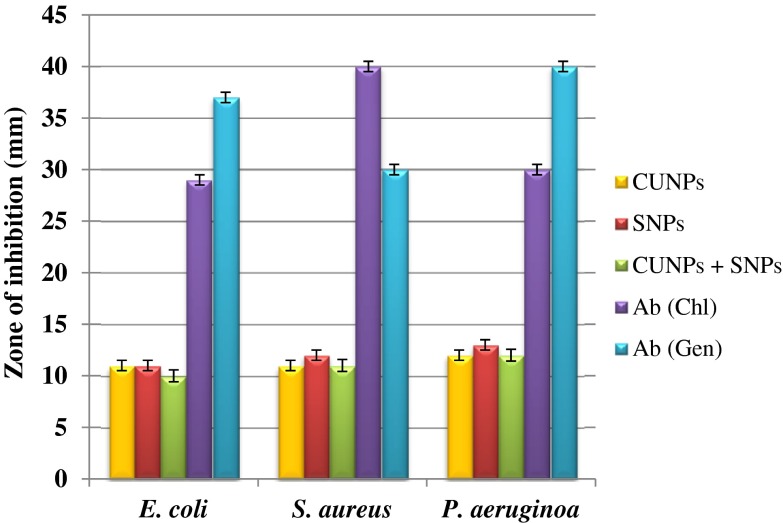
Antibacterial activity of Nano curcu against *E.coli*, *S.aureus*, *P.aeruginosa. CUNPs* Nano curcu, *SNPs* silver nanoparticles, *CUNPs+SNPs* Nano curcu+silver nanoparticles, *Ab (Chl)* antibiotic chloramphenicol, *Ab (Gen)* antibiotic gentamycin

**Fig. 8:**
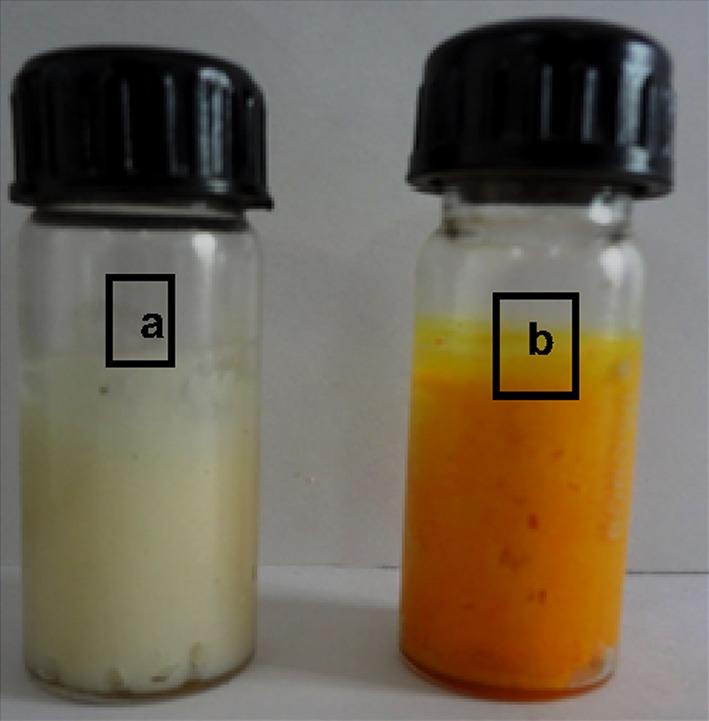
Formulated cream containing Nano curcu. *a* Control cream (without nanoparticles), *b* formulated cream (with nanoparticles)

**Fig. 9:**
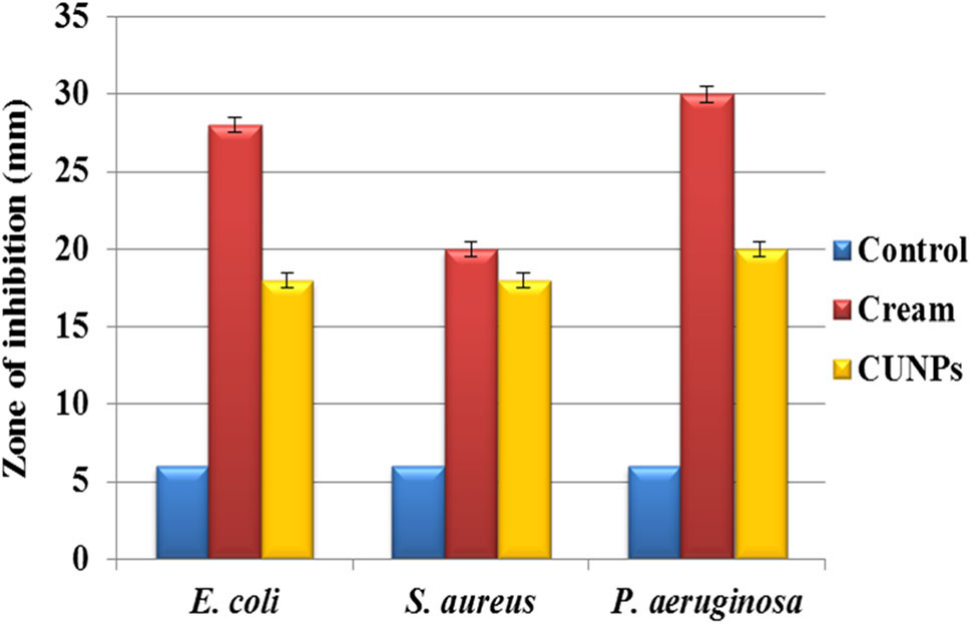
Antimicrobial activity of cream against pathogenic bacteria. *Control* cream without nanoparticles, *Cream* cream with nanoparticles, *CUNPs* Nano curcu

The antimicrobial activity of Nano curcu was tested against *E. coli* (ATCC 14948), *S. aureus* (ATCC 333591), and *P. aeruginosa* (MTCC 4676) (Fig. 7) by using Kierby–Bauer disc diffusion method. In the present study, Nano curcu, silver nanoparticles, combination of silver and Nano curcu, and antibiotics chloramphenicol and gentamycin were used to determine antibacterial activity. Nano curcu were found more effective (12 mm) against *P. aeruginosa*, whereas less effective (10 mm) against *S. aureus*. Silver nanoparticles were most effective against *P. aeruginosa* and less effective against *S. aureus*. Commercially available antibiotic chloramphenicol was found to be more effective against *S. aureus* (40 mm) and less effective against *E. coli* (29 mm)*. P. aeruginosa* was susceptible to antibiotic gentamycin, whereas *S. aureus* was less susceptible to gentamycin (Fig. 7). Formulated cream (Fig. 8) of Nano curcu was found to be much more effective against *P. aeruginosa* (30 mm) and less effective against *S. aureus* (20 mm) (Fig. 9).

## Discussion

To confirm the synthesis of Nano curcu, solubility testing was performed in which Nano curcu were soluble in water, while curcumin powder was not soluble in water (Fig. 1 inset). It confirms the synthesis of Nano curcu. The results of UV–visible spectroscopy obtained in our study, corroborate with the result of Alam et al. (2012) and Ghosh et al. (2011) which specifies the synthesis of Nano curcu. The findings of NTA are similar with results obtained by Montes et al. (2010) for determining the size of polystyrene nanoparticles. In FTIR analysis, peaks correspond to different functional groups. Among these, the absorption peak at 1626 cm^−1^ can be assigned for C=C stretching, 1452 cm^−1^ corresponds to C=H, and the absorption at 1146 cm^−1^ due to C–H stretching. The absorption peak at 1037 cm^−1^ might be due to C–N stretch. The absorption spectra of control might be attributed to the functional group such as benzene ring, C–O–C bond, and aromatic C–H stretching. These finding are supported by many researchers (Yadav et al. 2009; Yen et al. 2010; Sav et al. 2012). Zeta potential measurement is used to characterize the surface charge of Nano curcu. The nanoparticles in colloidal suspension or emulsion carry electric charge which may be positive or negative. The synthesized Nano curcu were moderately stable. The X-Ray diffraction pattern confirms the crystals of Nano curcu which correspond with the results obtained by Sav et al. (2012) and Yen et al. (2010). Antibacterial activity of Nano curcu and silver nanoparticles alone was found to be more or less similar. But when Nano curcu and silver nanoparticles both were used in combination, it showed less zone of inhibition as compared with silver and Nano curcu when it is tested singly against different bacteria. Gentamycin showed higher activity against Gram-negative bacteria, whereas chloramphenicol showed better action against Gram-positive bacteria. From in vitro antibacterial assay of Nano curcu, it was found that Nano curcu showed better antimicrobial activity against Gram-negative bacteria as compared to Gram-positive ones, whereas bulk curcumin is more effective against Gram-positive bacteria. The variation in activities among bacteria may reflect differences in cell wall structures and composition between Gram-negative and Gram-positive bacteria.

## Proposed mechanism of antimicrobial activity of Nano curcu

**Fig. 10:**
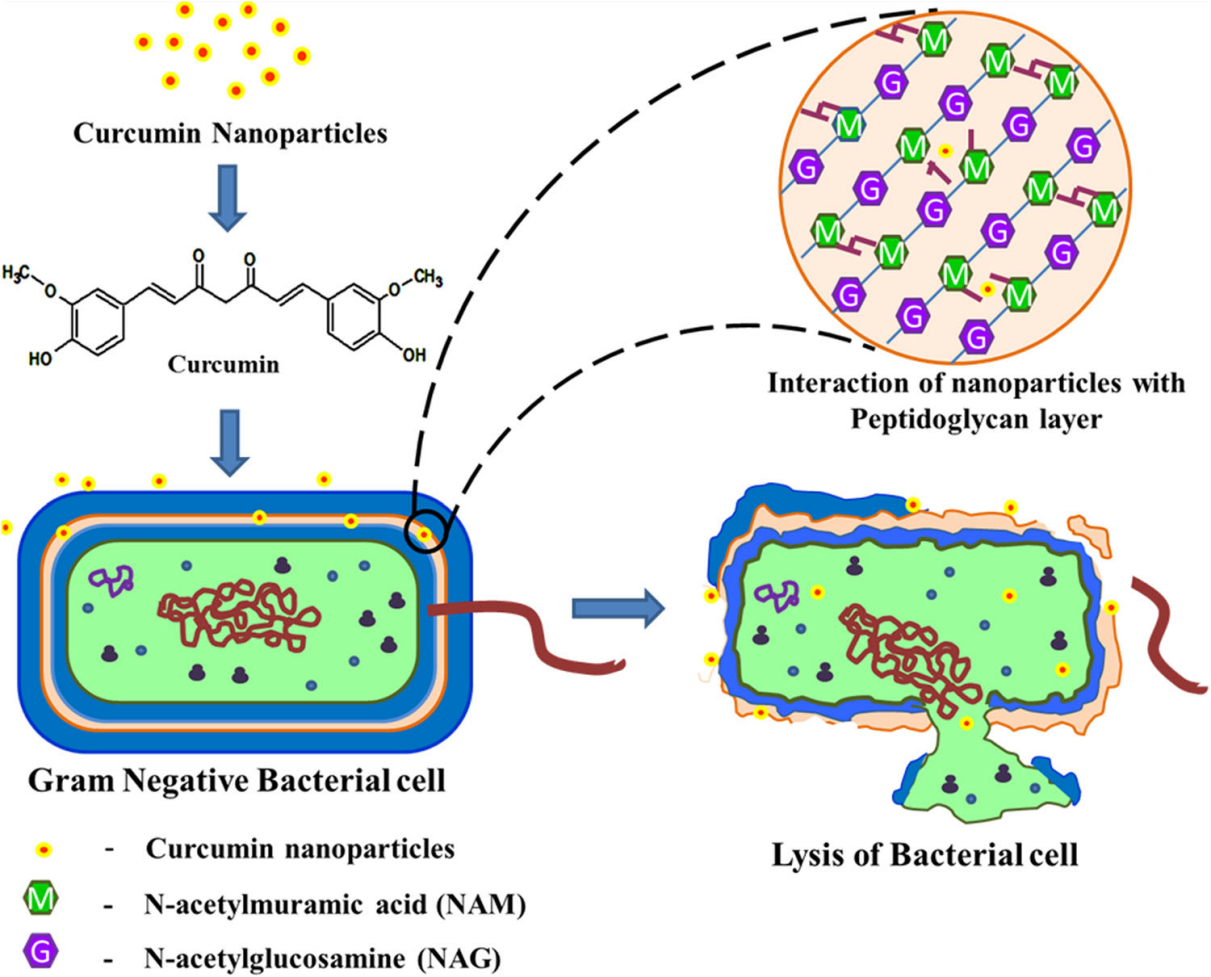
Proposed mechanism for antimicrobial activity of Nano curcu

The phenolic group in Nano curcu interacts with outer lipopolysaccharide layer present in the Gram-negative bacteria. Moreover, less peptidoglycan content helps in the weakening and breakage of bacterial cell wall, resulting in the death of the bacterial cell. In addition, antibacterial cream of Nano curcu possesses both the water phase and oil phase. Both the phases are stabilized by surfactant. The oil phase of the cream helps in the better interaction of Nano curcu with the outer lipopolysaccharide layer present in the Gram-negative bacteria, thereby causing the disruption of the less rigid cell wall of the Gram-negative bacteria (Fig. 10).

## Conclusions

Nano curcu were fabricated by physico-chemical method. It is simple, natural, and easy method used for the synthesis of Nano curcu. Synthesized nanoparticles showed its efficacy against bacteria, viz., *E. coli, S. aureus*, and *P. aeruginosa.* It can be concluded from the results that Nano curcu synthesized by sonication method inhibited activity of bacteria. The formulated cream is new generation of antiseptic cream, which could be used in the treatment of infection caused by *E. coli*, *S. aureus*, and *P. aeruginosa.* The Nano curcu could also be used for wound healing.
